# Detection and Tracking of NY-ESO-1-Specific CD8^+^ T Cells by High-Throughput T Cell Receptor β (TCRB) Gene Rearrangements Sequencing in a Peptide-Vaccinated Patient

**DOI:** 10.1371/journal.pone.0136086

**Published:** 2015-08-20

**Authors:** Manami Miyai, Shingo Eikawa, Akihiro Hosoi, Tamaki Iino, Hirokazu Matsushita, Midori Isobe, Akiko Uenaka, Heiichiro Udono, Jun Nakajima, Eiichi Nakayama, Kazuhiro Kakimi

**Affiliations:** 1 Department of Immunotherapeutics, The University of Tokyo Hospital, Bunkyo-ku, Tokyo, Japan; 2 Department of Immunology, Okayama University Graduate School of Medicine, Dentistry and Pharmaceutical Sciences, Okayama, Okayama, Japan; 3 Faculty of Health and Welfare, Kawasaki University of Medical Welfare, Kurashiki, Okayama, Japan; 4 Department of Thoracic Surgery, The University of Tokyo Hospital, Bunkyo-ku, Tokyo, Japan; Mie University Graduate School of Medicine, JAPAN

## Abstract

Comprehensive immunological evaluation is crucial for monitoring patients undergoing antigen-specific cancer immunotherapy. The identification and quantification of T cell responses is most important for the further development of such therapies. Using well-characterized clinical samples from a high responder patient (TK-f01) in an NY-ESO-1f peptide vaccine study, we performed high-throughput T cell receptor β-chain (TCRB) gene next generation sequencing (NGS) to monitor the frequency of NY-ESO-1-specific CD8^+^ T cells. We compared these results with those of conventional immunological assays, such as IFN-γ capture, tetramer binding and limiting dilution clonality assays. We sequenced human TCRB complementarity-determining region 3 (CDR3) rearrangements of two NY-ESO-1f-specific CD8^+^ T cell clones, 6-8L and 2F6, as well as PBMCs over the course of peptide vaccination. Clone 6-8L possessed the TCRB CDR3 gene TCRBV11-03*01 and BJ02-01*01 with amino acid sequence CASSLRGNEQFF, whereas 2F6 possessed TCRBV05-08*01 and BJ02-04*01 (CASSLVGTNIQYF). Using these two sequences as models, we evaluated the frequency of NY-ESO-1-specific CD8^+^ T cells in PBMCs ex vivo. The 6-8L CDR3 sequence was the second most frequent in PBMC and was present at high frequency (0.7133%) even prior to vaccination, and sustained over the course of vaccination. Despite a marked expansion of NY-ESO-1-specific CD8^+^ T cells detected from the first through 6th vaccination by tetramer staining and IFN-γ capture assays, as evaluated by CDR3 sequencing the frequency did not increase with increasing rounds of peptide vaccination. By clonal analysis using 12 day in vitro stimulation, the frequency of B*52:01-restricted NY-ESO-1f peptide-specific CD8^+^ T cells in PBMCs was estimated as only 0.0023%, far below the 0.7133% by NGS sequencing. Thus, assays requiring in vitro stimulation might be underestimating the frequency of clones with lower proliferation potential. High-throughput TCRB sequencing using NGS can potentially better estimate the actual frequency of antigen-specific T cells and thus provide more accurate patient monitoring.

## Introduction

To assess the efficacy of cancer immunotherapy, identification and quantification of antigen-specific T cell responses during the course of treatment is required [1,2]. In general, monitoring techniques such as tetramer binding, intracellular cytokine staining, cytokine capture assays or ELISPOT are used singly or combined to measure immune reactivity. Each assay has its own particular advantages and disadvantages. Ideally, direct ex vivo monitoring is desired, but the frequency of antigen-specific T cells is commonly below the level of detection of these assays. Therefore, in vitro stimulation may be required to expand the antigen-specific T cells prior to their measurement [1].

To date, ELISPOT is the most sensitive assay commonly applied for ex vivo analysis. However, detection depends on cytokine production and therefore T cells that lack this ability cannot be detected. The tetramer binding assay can be applied only when there is an appropriate match between known patient HLA type and CTL epitope and available tetramers [3,4] and because it is flow cytometry-based, sensitivity is an issue. Generally, 0.1% of antigen-specific T cells in the sample is required for optimal analysis and therefore an in vitro stimulation step may be needed to reliably perform this assay. Intracellular cytokine assays [5] and cytokine capture assays [6] are also flow cytometry-based and similar to ELISPOT both depend on cytokine production by the T cells. The sensitivity of both assays is comparable to that of the tetramer assay. Therefore, to determine frequencies of antigen-specific T cells, greater sensitivity is desirable in assays not depending on cytokine production or any in vitro expansion that could cause bias by selecting for T cells with greater proliferative potential.

Recently, advances in next generation sequencing (NGS) technologies have been applied to T cell receptor (TCR) repertoire analysis [7]. High-throughput sequencing with single clonotype resolution for estimating clonal diversity [8], tracking minimal residual disease in blood cancers [9,10] and multiple clones simultaneously has been developed. A repertoire-wide assessment of T cell responses by high-throughput TCR sequencing has also been used to track T cell immune responses following immunomodulatory cancer therapy [11–13]. Here, we applied high-throughput T cell receptor β-chain (TCRB) gene NGS to quantify antigen-specific CD8^+^ T cells ex vivo which were present at frequencies that were low or undetectable by more traditional methods, and to monitor them over the course of NY-ESO-1f peptide vaccination [14]. We also compare the different methodologies that have been used to evaluate the frequency of vaccine-induced antigen-specific T cells with TCRB CDR3 NGS and consider the challenges and opportunities for the field.

## Materials and Methods

### Patient TK-f01

**Fig 1:**
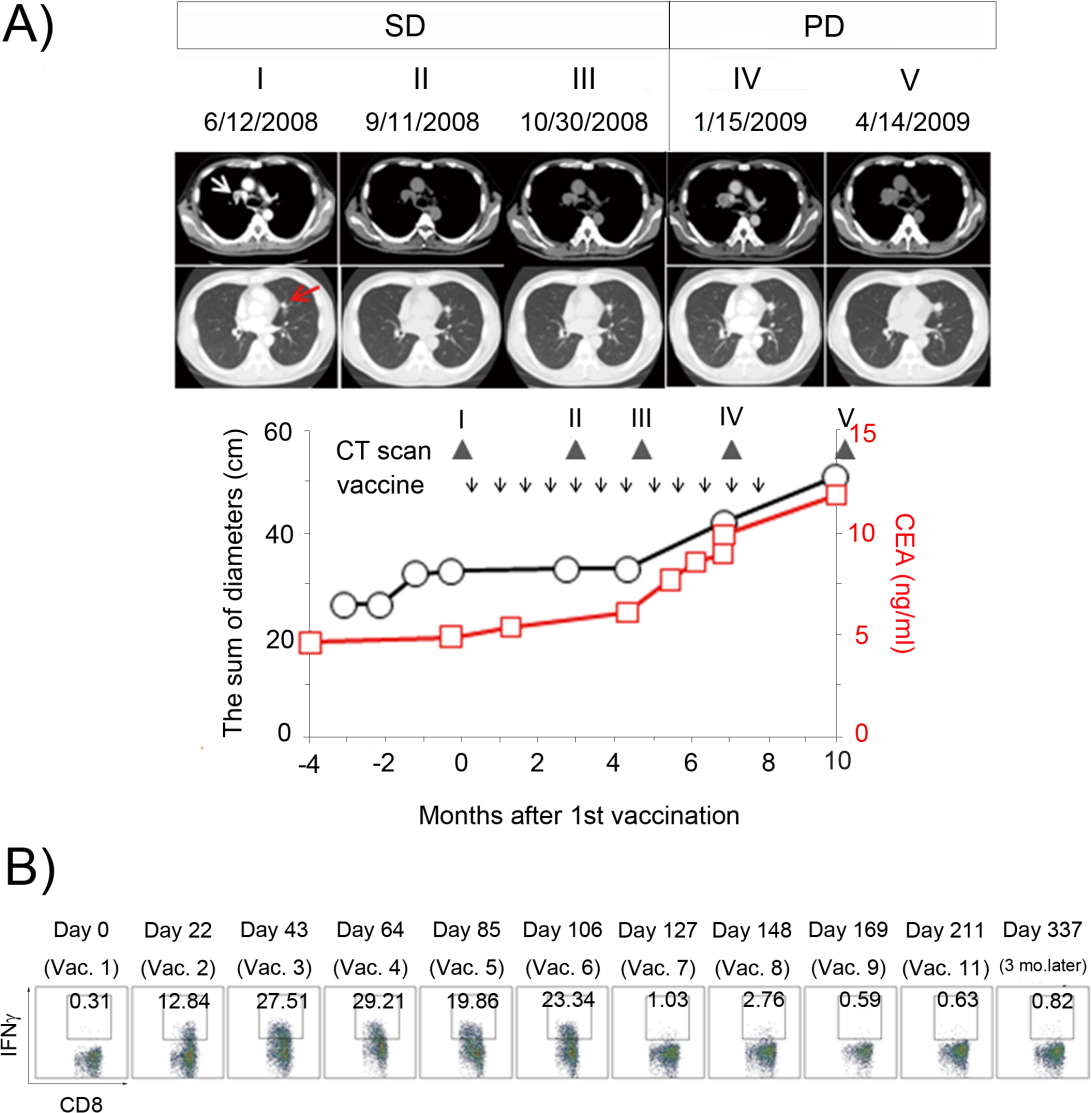
Clinical and immunological response to NY-ESO-1f peptide vaccine in patient TK-f01. **(A) Clinical response of patient TK-f01.** A right hilar lymph node (white arrow) and a small metastatic nodule in the left lobe (red arrow) were monitored on CT images as target lesions. The sum of diameters of tumor lesions (black line) and serum CEA levels (red line) are plotted. Vaccination (arrow) and CT scan (triangle) are indicated. **(B) NY-ESO-1-specific CD8** ^**+**^ **T response.** PBMCs were obtained at the indicated time points during vaccination. CD8^+^ T cells were purified from PBMCs using magnetic beads (Miltenyi Biotec) and stimulated with NY-ESO-1 overlapping peptide pools in vitro for 12 days. The IFN-γ capture assay was then performed 4 hr after stimulation with PFA-treated CD4- and CD8-depleted PBMC pulsed with the peptides. The net percentage of IFN-γ-producing cells within total CD8^+^ T cells was determined.

Lung cancer patient TK-f01 received a right middle lobectomy in October 2004, followed by postoperative adjuvant chemotherapy with Tegafur-Uracil (UFT) for 6 months. In April 2007, a CT scan detected recurrence in the left lung and in a right hilar lymph node. Although the patient received three courses of combination chemotherapy with carboplatin and paclitaxel, the tumor grew progressively. He was then enrolled in a phase I clinical trial of NY-ESO-1f peptide vaccine in June 2008. The study design using the NY-ESO-1f peptide (NY-ESO-1 91–110: YLAMPFATPMEAELARRSLA) was described previously [14]. The protocol was approved by the Ethics Committee of the University of Tokyo (ID: 1935-(2)) according to the Declaration of Helsinki. Written informed consent was obtained before enrollment. The study was conducted according to Good Clinical Practice guidelines and was registered in the University Hospital Medical Information Network Clinical Trials Registry (UMIN-CTR) Clinical Trial (Unique trial number: UMIN000001260) on July 24, 2008 (UMIN-CTRURL: http://www.umin.ac.jp/ctr/index.htm). As we reported previously [14], tumor growth was suppressed after initiation of NY-ESO-1f peptide vaccination (Fig 1A). The response was evaluated as SD at the end of the sixth vaccination; he subsequently received another cycle of six vaccinations. However, the tumor started to grow again after the eighth vaccination, paralleled by accelerated elevation in the serum CEA level. A >20% increase in the sum of target lesion diameters was detected after the 11th vaccination, evaluated as progressive disease (PD).

### Blood samples

Peripheral blood was drawn before vaccination, at each immunization and 4 weeks and 3 months after the last immunization. Peripheral blood mononuclear cells (PBMCs) and plasma were isolated by density gradient centrifugation using Lymphoprep (Axis Shield PoC AS, Oslo, Norway). CD8^+^ T cells were purified from PBMCs using magnetic beads (Miltenyi Biotec, Auburn, CA) and stored in liquid N_2_ as described previously [14,15]. The stored samples were thawed and used in this study.

### IFN-γ capture assay

Frozen cells were thawed and resuspended in AIM-V medium (Invitrogen, Carlsbad, CA) supplemented with 5% heat-inactivated pooled human serum and kept at room temperature for 2 hr. CD8^+^ T cell-enriched populations (2 × 10^6^) were cultured for 12 days in the presence of 28 18-mer overlapping peptides and a 30-mer C-terminal peptide spanning the entire NY-ESO-1 protein (1 μg/ml for each peptide) as described previously [14,15]. The IFN-γ capture assay [16,17] was carried out according to the manufacturer's protocol (Miltenyi Biotec) as described previously [14,15].

### Cloning of CD8^+^ T cells, tetramer construction and staining

NY-ESO-1-specific CD8^+^ T cell clones were established by limiting dilution after in vitro stimulation as described in detail previously [15]. Peptide/HLA tetramers were produced as described [3,15,18]. NY-ESO-1 92-100/B*35:01 and NY-ESO-1 94-104/B*35:01 tetramers were used. The HIV Env/A*24:02 tetramer was used as a negative control. For staining, cells were incubated with tetramer at a concentration of 20 μg/ml for 15 min at 37°C, followed by incubation with a FITC-conjugated anti-CD8 mAb (Miltenyi Biotec) on ice for 15 min, and then analyzed on a FACS Canto II (Becton Dickinson).

### Analysis of human TCRB complementarity-determining region 3 (CDR3) rearrangements

Genomic DNA extraction was performed using the AllPrep DNA/RNA mini Kit (Qiagen). The TCRB CDR3 region was amplified and sequenced from a standardized quantity of DNA using the ImmunoSEQ assay (Adaptive Biotechnologies, Seattle, WA) [7,19,20]. Briefly, a multiplex PCR system was used to amplify the rearranged TCRB CDR3 sequences from DNA. The 87 base-pair fragment is sufficient to identify the VDJ region spanning each unique TCRB CDR3. Amplicons were sequenced using the Illumina platform. TCRβ V, D and J gene definitions were provided by the IMGT database (www.imgt.org). The assay is quantitative, having utilized a complete synthetic repertoire of TCRs to establish an amplification baseline and adjust the assay chemistry to correct for primer bias. In addition, bar-coded, spiked-in synthetic templates were used to measure sequencing coverage and residual PCR bias. This information is used for further PCR bias correction and the estimation of input amounts of sequence-able templates. The resulting data is filtered and clustered using both the relative frequency ratio between similar clones and a modified nearest-neighbor algorithm to merge closely related sequences and remove both PCR and sequencing errors. Data was analyzed using the ImmunoSEQ analyzer toolset. The reproducibility of the assay was shown in S1 Fig.

## Results and Discussion

### Clinical course of patient TK-f01 and evaluation of his NY-ESO-1-specific CD8^+^ T cell responses by the IFN-γ capture assay

We conducted a phase I clinical trial of a 20-mer NY-ESO-1f peptide vaccine (NY-ESO-1 91–110) and have already reported that vaccination with this peptide resulted in an increase or induction of NY-ESO-1 antibody responses, as well as CD4^+^ and CD8^+^ T cell responses [14,15]. Patient TK-f01 was a high responder; a robust and sustained CD8^+^ T cell response to NY-ESO-1 was detected after initiating vaccination [14,15]. As shown in Fig 1, patient TK-f01´s disease was well-controlled and the tumor remained stable for several months, classed as SD at the end of the sixth vaccination. However, serum CEA level started to rise with increased diameters of target lesions thereafter (Fig 1A).

As we reported previously [14], the IFN-γ capture assay was performed to monitor NY-ESO-1f-specific CD8^+^ T cell responses in this patient (Fig 1B). However, their frequency was below the level of detection by this assay ex vivo. Therefore, CD8^+^ T cells had to be enriched from PBMC and cultured for 12 days with irradiated autologous CD4- and CD8-depleted PBMC in the presence of overlapping NY-ESO-1 peptides. These in vitro stimulated cells were then tested for NY-ESO-1-reactive IFN-γ secretion (Fig 1B). A vigorous CD8 T-cell response was observed even after the first vaccination (day 22), sustained until after the fifth vaccination (day 106). Suddenly, the response became undetectable on day 127 (after 6 vaccinations) and remained so thereafter. The disappearance of NY-ESO-1-specific CD8^+^ T cell responses was paralleled by disease progression.

### Amplification and sequencing of TCRB CDR3 regions

To determine the frequency of NY-ESO-1-specific CD8^+^ T cells and monitor dynamic changes on vaccination without recourse to in vitro expansion cultures, we performed NGS-based high-throughput TCRB CDR3 sequencing of cryopreserved PBMCs from TK-f01 to identify and track NY-ESO-1-specific T cell clones. The TCRB CDR3 gene sequences enabled clonality and repertoire analysis of adaptive immunity at single clonotype resolution to be undertaken; however, CDR3 sequencing cannot identify antigen specificity. Therefore, we first determined the CDR3 sequence of two NY-ESO-1-specific CD8^+^ T cell clones, the HLA-B35:01-restricted clone 2F6 and the HLA-B52:01-restricted clone 6-8L, which were previously established [15]. Sequences from 2F6 and 6-8L were subsequently used as references to identify and quantify the frequency of NY-ESO-1-specific CD8^+^ T cells in PBMCs over the course of vaccination.

**Fig 2:**
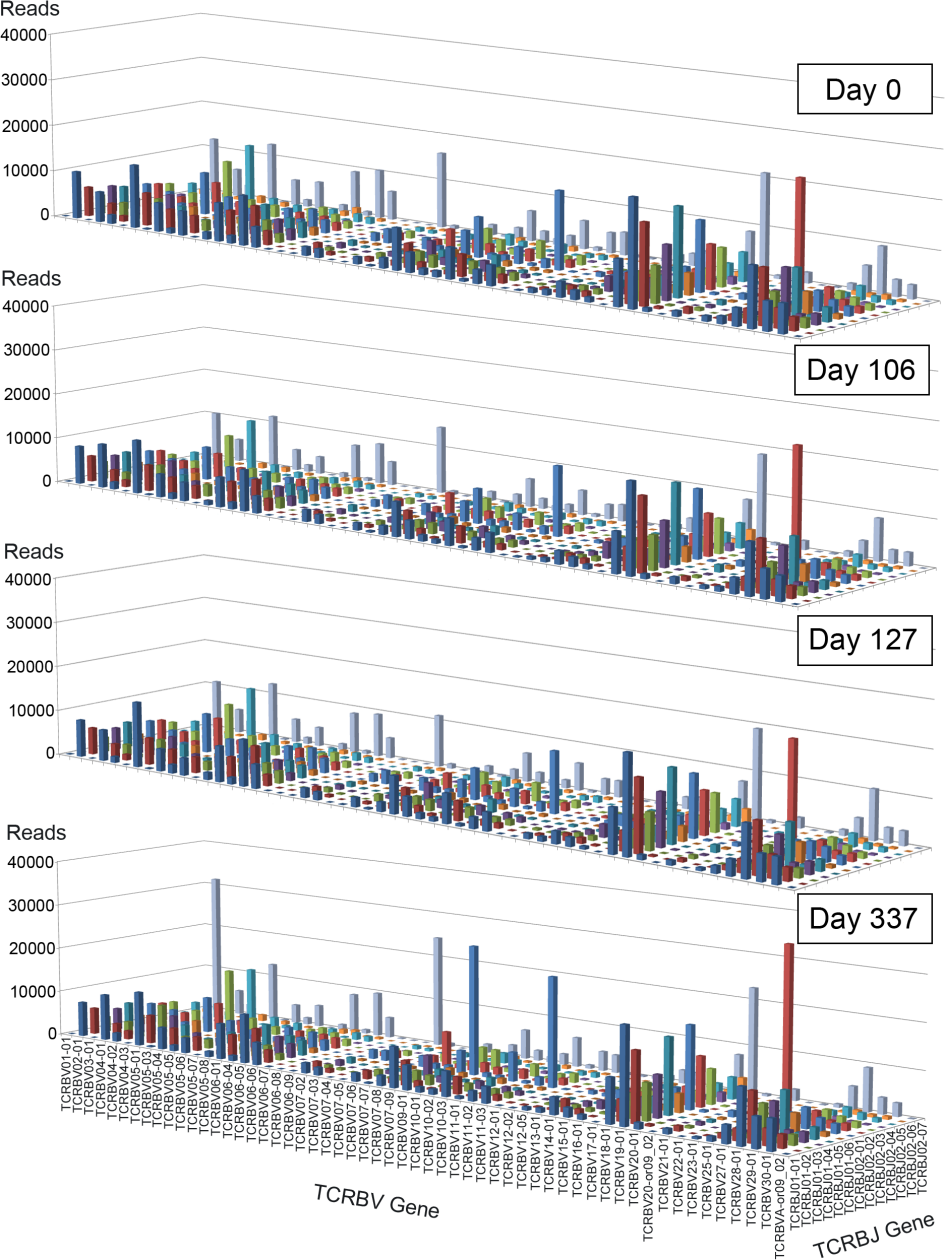
TCRB CDR3 repertoire analysis of PBMCs over the course of NY-ESO-1f peptide vaccination. Differential usages of TCRBV and BJ genes in the patient’s PBMCs at days 0, 106, 127 and 337 are shown. Read numbers of V-J gene pair are shown in the 3D graph.

In the first set of NGS analysis, we obtained on average 2.42×10^6^ sequence reads from the two NY-ESO-1-specifc CD8^+^ T clones and 1.84±0.096×10^6^ sequence reads from PBMC; of these 1.83×10^6^ were productive and 0.59×10^6^ were non-productive (CD8^+^ T clones) and 1.54±0.082×10^6^ were productive and 0.30±0.016×10^6^ were non-productive (PBMC) (Table 1). There were 593 and 379 unique productive TCRB CDR3 sequences from 6-8L and 2F6, respectively, contrasting with 5.99±0.74×10^4^ unique productive sequences from PBMCs. We also performed second NGS analysis with less total reads and obtained equivalent results (Table 1, 2nd run). The TCRB repertoires were very diverse in the patient’s PBMCs and the physiological fluctuation of TCR repertoire was depicted in Fig 2. Similar frequency distributions and no biased BV- or BJ-gene usage in PBMC were observed over the course of vaccination. In both pre-vaccination and post-vaccination samples, no skewed distribution or changes of TCRBV and BJ gene segment usage were observed, suggesting that the impact of vaccine-induced NY-ESO-1-specific CD8^+^ T cells on the entire TCR repertoire of the patient’s PBMCs was negligible.

**Table 1:** Summary of TCRB sequencing.

		**Sequences**					**Unique sequence**				
		**Total reads**	**Productive (reads)**	**Productive (%)**	**Non productive (reads)**	**Non productive (%)**	**Total reads**	**Productive (reads)**	**Productive (%)**	**Non productive (reads)**	**Non productive (%)**
**CTL clones**	**6-8L**	1889744	1778683	94.12	111061	5.88	788	593	75.25	195	24.75
	**2F6**	2943536	1878749	63.83	1064787	36.17	573	379	66.14	194	33.86
	**Average**	2416640	1828716	78.98	587924	21.03	681	486	70.70	195	29.31
		**Sequences**					**Unique sequence**				
		**Total reads**	**Productive (reads)**	**Productive (%)**	**Nonproductive (reads)**	**Non productive (%)**	**Total reads**	**Productive (reads)**	**Productive (%)**	**Non productive (reads)**	**Non productive (%)**
**PBMCs**	**Day 0**	1950950	1626108	83.35	324842	16.65	72597	59296	81.68	13301	18.32
	**Day 22**	1856996	1542908	83.09	314088	16.91	68590	56079	81.76	12511	18.24
	**Day 43**	1713666	1436563	83.83	277103	16.17	71321	58420	81.91	12901	18.09
	**Day 106**	1739425	1450175	83.37	289250	16.63	74477	60991	81.89	13486	18.11
	**Day 127**	1820734	1525227	83.77	295507	16.23	67768	55481	81.87	12287	18.13
	**Day 148**	1777708	1489997	83.82	287711	16.18	74928	61310	81.83	13618	18.17
	**Day 232**	1927013	1618652	84.00	308361	16.00	93169	76404	82.01	16765	17.99
	**Day 337**	1959759	1651186	84.25	308573	15.75	62780	51378	81.84	11402	18.16
	**Average**	1843281	1542602	83.69	300679	16.32	73204	59920	81.85	13284	18.15
	**SD**	96179	82292	0.38	15885	0.38	8997	7415	0.10	1583	0.10
		**Sequences**					**Unique sequence**				
		**Total reads**	**Productive (reads)**	**Productive (%)**	**Nonproductive (reads)**	**Non productive (%)**	**Total reads**	**Productive (reads)**	**Productive (%)**	**Non productive (reads)**	**Non productive (%)**
**PBMCs (2nd run)**	**Day 64**	765929	644167	84.10	121762	15.90	75334	62089	82.42	13245	17.58
	**Day 337**	645391	549334	85.12	96057	14.88	53887	44389	82.37	9498	17.63
	**Average**	705660	596751	84.61	108910	15.00	64611	53239	82.40	11372	17.61

### High-throughput sequencing of the TCRB CDR3 regions of NY-ESO-1f-specific clones

**Fig 3:**
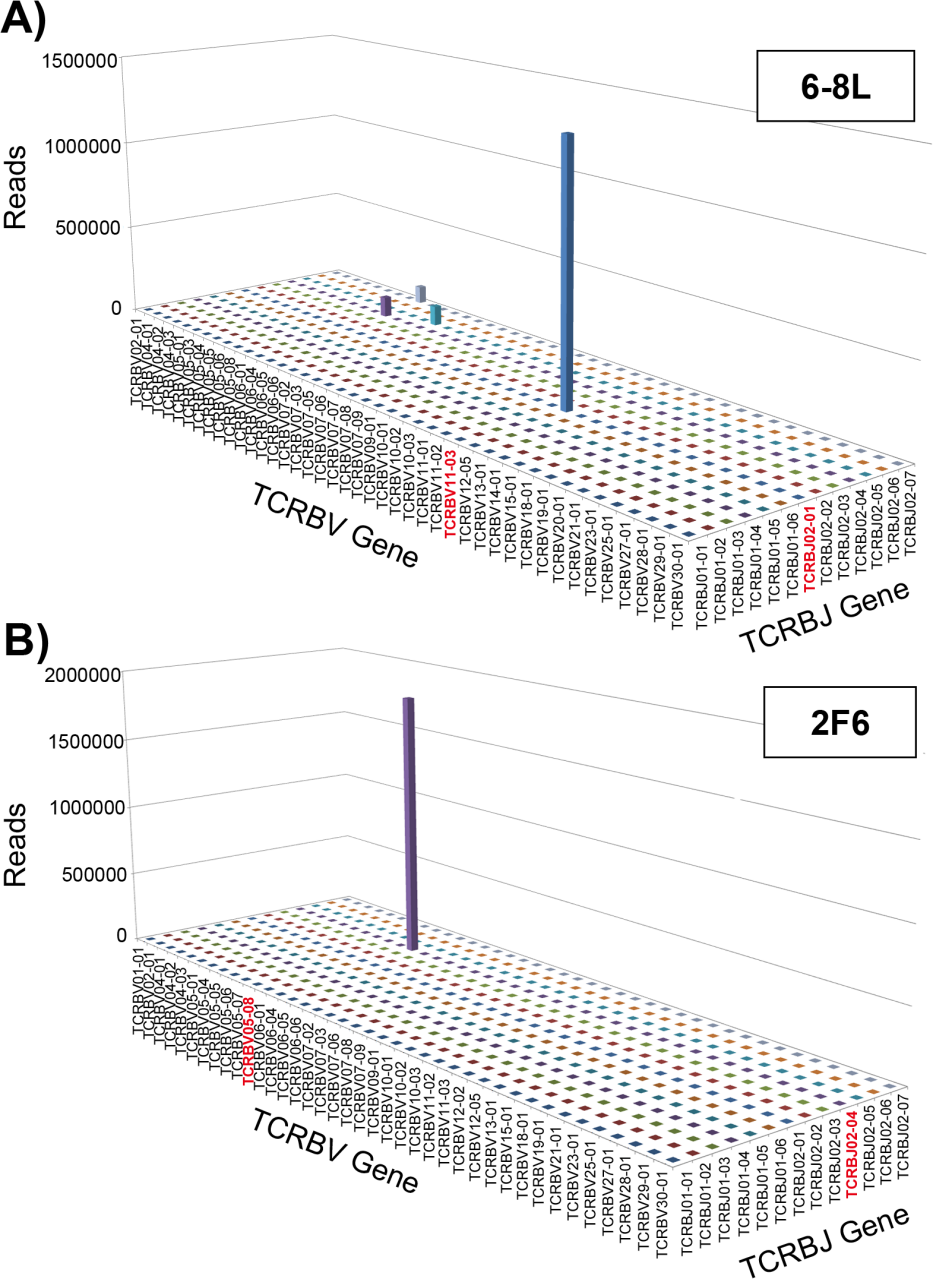
TCRBV and BJ usage of NY-ESO-1-specific CD8^+^ T cell clones. TCRB CDR3 sequence analyses of NY-ESO-1-specific CD8^+^ T cell clones were performed. (A) HLA-B52:01-restricted clone 6-8L has the dominant V-J pair TCRBV11-03*01 and TCRBJ02-01*01. Only a few V-J pairs other than this dominant pair were observed in clone 6-8L. (B) HLA-B35:01-restricted clone 2F6 has a dominant V-J pair TCRBV05-08*01 and TCRBJ02-04*01.

The NY-ESO-1-specific CD8^+^ T cell clones 6-8L and 2F6 had been established by limiting dilution from this patient´s PBMC on day 64, which was 21 days after the 3rd vaccination and just prior to the 4th vaccination [15]. Although a few other TCRBV gene segments and J gene segments were detected, most 6-8L CDR3 was TCRBV11-03*01 and BJ02-01*01 with amino acid sequence CASSLRGNEQFF (Fig 3A). TCRB of 2F6 was TCRBV05-08*01 and BJ02-04*01 with amino acid sequence CASSLVGTNIQYF (Fig 3B); no other TCRBV family segments or J gene segments were detected. These results also confirm the clonality of 6-8L and 2F6 cells.

### Tracking NY-ESO-1-specific CD8^+^ T cells in PBMCs from patient TK-f01

**Fig 4:**
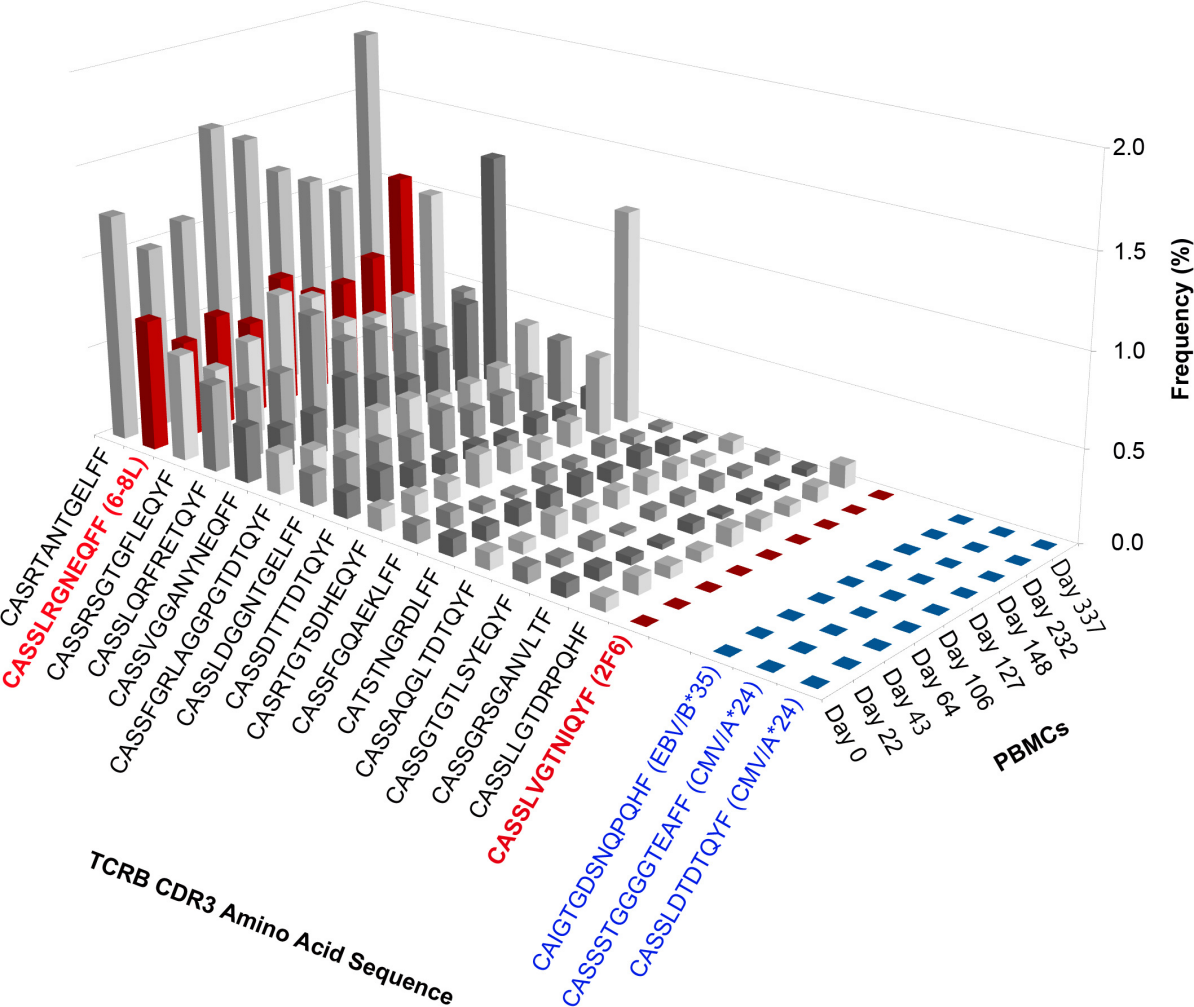
TCRB repertoire analysis for tracking NY-ESO-1-specific CD8^+^ T cell clones. The frequencies of the 15 most frequent TCRB CDR3 sequences in pretreatment PBMCs (Day 0) and from the NY-ESO-1-specific clones 6-8L and 2F6 were tracked over time during the course of treatment. The known TCRB CDR3 sequences for HLA-B*35-restricted EBV- and HLA-A*24-restricted CMV-specific T cells were also detected in PBMCs. The frequency of each CDR3 sequence is shown. The CDR3 sequence of clone 6-8L was the second most frequent sequence and remained present at high frequency from the initiation of the treatment to three months after the last vaccination. The CDR3 sequence of 2F6 was not detected in PBMCs on day 0; however, it was detected at low frequency on day 106 and day 337.

We asked whether the identified TCRB CDR3 sequences matching either 6-8L or 2F6 were detected in PBMCs, so as to monitor antigen-specific TCRs. In addition, the 15 most frequent TCRB sequences in PBMCs and known TCRB CDR3 sequences of EBV- or CMV-specific T cells [21,22] are listed in Table 2 to evaluate the physiological fluctuation. The more detailed list is provided as S1 Table. These most frequent clones present in blood at baseline either increased or decreased over the course of treatment (Fig 4). EBV- or CMV-specific clones were detected in patient’s PBMC intermittently; no impact of NY-ESO-1f vaccination on these clones was observed (Fig 4).

**Table 2:** The 15 most frequent CDR3 sequences in the pretreatment PBMCs and dominant CDR3 sequences of NY-ESO-1-specific CD8 T cell clones.

			Day 0		Day 22		Day 43		Day 64		Day 106		Day 127		Day 148		Day 232		Day 337		Day 337 (2nd)	
			Rank	Counts (reads)	Rank	Counts (reads)	Rank	Counts (reads)	Rank	Counts (reads)	Rank	Counts (reads)	Rank	Counts (reads)	Rank	Counts (reads)	Rank	Counts (reads)	Rank	Counts (reads)	Rank	Counts (reads)
Amino acid	TCRBV	TCRBJ		Frequency (%)		Frequency (%)		Frequency (%)		Frequency (%)		Frequency (%)		Frequency (%)		Frequency (%)		Frequency (%)		Frequency (%)		Frequency (%)
CASRTANTGELFF	TCRBV25-01	TCRBJ02-02	1	24173	1	18538	1	18832	1	11951	1	25141	1	22127	1	19595	1	19146	1	36338	1	17257
				1.239		0.9983		1.0989		1.5603		1.4454		1.2153		1.1023		0.9936		1.8542		2.6739
**CASSLRGNEQFF**	**TCRBV11-03**	**TCRBJ02-01**	**2**	**13917**	**2**	**9825**	**2**	**10515**	**4**	**3897**	**2**	**12267**	**2**	**10146**	**2**	**9767**	**2**	**12386**	**4**	**20675**	**5**	**6166**
**(6-8L)**				**0.7133**		**0.5291**		**0.6136**		**0.5088**		**0.7052**		**0.5572**		**0.5494**		**0.6428**		**1.055**		**0.9554**
CASSRSGTGFLEQYF	TCRBV07-02	TCRBJ02-07	3	11353	3	8040	3	8993	2	5558	3	11243	4	8025	3	7193	4	8849	5	19735	4	11185
				0.5819		0.433		0.5248		0.7257		0.6464		0.4408		0.4046		0.4592		1.007		1.7331
CASSLQRFRETQYF	TCRBV05-01	TCRBJ02-05	4	9263	4	6980	5	6938	3	5080	4	7867	3	8189	4	6249	6	6248	6	9487	6	5379
				0.4748		0.3759		0.4049		0.6632		0.4523		0.4498		0.3515		0.3242		0.4841		0.8334
CASSVGGANYNEQFF	TCRBV09-01	TCRBJ02-01	5	5931	8	4197	6	4036	5	2804	5	4978	5	4024	5	5522	3	10095	2	25655	2	13977
				0.304		0.226		0.2355		0.3661		0.2862		0.221		0.3106		0.5239		1.3091		2.1657
CASSFGRLAGGPGTDTQYF	TCRBV05-01	TCRBJ02-03	6	4356	11	3123	8	3223	6	1828	6	4118	6	3243	7	3276	7	3991	7	7673	7	3587
				0.2233		0.1682		0.1881		0.2387		0.2367		0.1781		0.1843		0.2071		0.3915		0.5558
CASSLDGGNTGELFF	TCRBV07-07	TCRBJ02-02	7	3432	9	3374	7	3373	8	1161	7	3998	7	2893	8	3060	8	3787	8	7009	8	1825
				0.1759		0.1817		0.1968		0.1516		0.2298		0.1589		0.1721		0.1965		0.3576		0.2828
CASSDTTTDTQYF	TCRBV06-04	TCRBJ02-03	8	2711	10	3343	11	1993	12	725	12	1712	11	1383	11	1836	11	1606	12	2444	19	609
				0.139		0.18		0.1163		0.0947		0.0984		0.076		0.1033		0.0833		0.1247		0.0944
CASRTGTSDHEQYF	TCRBV02-01	TCRBJ02-07	9	1914	16	2081	12	1782	7	1395	9	2383	8	1829	9	2504	5	8401	3	23313	3	11600
				0.1177		0.1121		0.104		0.1821		0.137		0.1005		0.1409		0.436		1.1896		1.7974
CASSFGQAEKLFF	TCRBV28-01	TCRBJ01-04	10	2297	24	1655	24	812	53	205	15	1417	20	902	14	1422	20	914	51	597	428	52
				0.0981		0.0891		0.0474		0.0268		0.0815		0.0495		0.08		0.0474		0.0305		0.0081
CATSTNGRDLFF	TCRBV15-01	TCRBJ01-04	11	1885	28	1497	13	1706	10	739	11	1900	12	1359	12	1743	15	1262	78	435	45	226
				0.0966		0.0806		0.0996		0.0965		0.1092		0.0746		0.098		0.0655		0.0222		0.0350
CASSAQGLTDTQYF	TCRBV07-09	TCRBJ02-03	12	1811	33	1237	10	2128	13	653	14	1673	9	1796	13	1615	19	939	21	1445	30	382
				0.0928		0.0666		0.1242		0.0853		0.0962		0.0986		0.0908		0.0487		0.0737		0.0592
CASSGTGTLSYEQYF	TCRBV19-01	TCRBJ02-07	13	1677	45	904	18	1033	72	171	20	1015	18	1089	15	1387	21	804	36	975	138	104
				0.086		0.0487		0.0603		0.0223		0.0584		0.0598		0.078		0.0417		0.0498		0.0161
CASSGRSGANVLTF	TCRBV06-04	TCRBJ02-06	14	1503	32	1265	29	742	48	222	21	1000	39	441	24	692	27	689	39	761	132	107
				0.077		0.0681		0.0433		0.0290		0.0575		0.0242		0.0389		0.0358		0.0388		0.0166
CASSLLGTDRPQHF	TCRBV28-01	TCRBJ01-05	15	1420	21	1815	17	1087	21	448	13	1701	13	1229	18	1088	12	1576	13	2442	14	723
				0.0728		0.0977		0.0634		0.0585		0.0978		0.0675		0.0612		0.0818		0.1246		0.1120
**CASSLVGTNIQYF**	**TCRBV05-08**	**TCRBJ02-04**		**0**		**0**		**0**		**0**	**19828**	**13**		**0**		**0**		**0**	**2677**	**62**		**0**
**(2F6)**				**0**		**0**		**0**		**0**		**0.0007**		**0**		**0**		**0**		**0.0032**		**0**
public TCR																						
CAIGTGDSNQPQHF	TCRBV10-03	TCRBJ01-05		0		0	21433	11		0		0		0	665	88	3480	44		0		0
(EBV specific TCR / B*35)				0		0		0.0006		0		0		0		0.005		0.0023		0		0
CASSSTGGGGTEAFF	TCRBV07-03	TCRBJ01-01		0	19529	17		0		0		0		0		0	18270	20	8693	34	18810	6
(CMV specific TCR / A*24)				0		0.0009		0		0		0		0		0		0.0010		0.0017		0.0009
CASSLDTDTQYF	TCRBV05-06	TCRBJ02-03		0	16055	21	10117	28	16085	9		0		0		0		0		0		0
(CMV specific TCR / A*24)				0		0.0011		0.0016		0.0012		0		0		0		0		0		0
Total reads				1950950		1856996		1713666		765929		1739425		1820734		1777708		1927013		1959759		645391

Bold: CDR3 sequence of NY-ESO-1-specific CTL clones

Clones with sequences identical to the 6-8L amino acid sequence CASSLRGNEQFF were the second most frequently detected in PBMC (Table 2). It is noteworthy that this clone was already present at high frequency prior to vaccination. Thus, the frequency of the 6-8L clonal sequence was 0.7133% before vaccination, 0.529% after the first vaccination, 0.5572 after 6th vaccination, 0.549% at the 11th vaccination and 1.055% at 3 months after the last (11th) vaccination. These results show that NY-ESO-1f-specific CD8^+^ T cell clone 6-8L sequence was present in patient PBMC prior to peptide vaccination and was maintained over the course of treatment. Hence, vaccination did not change the frequency of this particular clone. In contrast, sequences identical to the 2F6 amino acid sequence CASSLRGNEQFF were detected in PBMC neither prior to vaccination, nor 22, 43 and 64 days. However, they became detectable at day 106, after the 5th vaccination, becoming undetectable again at days 127, 148 and 238. They became detectable once more at day 337, 3 months after the last vaccination (Table 2). The frequency was only 0.0007% at day 106 and 0.0032% at day 337. Despite these fluctuations, it is clear that NY-ESO-1f peptide vaccination did not affect either the high frequency clone (6-8L) or low frequency clone (2F6) sequences. These results are therefore completely at odds with our previous observations from the IFN-γ capture assay and tetramer analysis, both using in vitro expanded CD8^+^ T cells, which had concluded that NY-ESO-1f peptide vaccination rapidly expanded NY-ESO-1f-specific CD8^+^ T cells which suddenly disappeared during the course of vaccination.

### Tetramer staining, IFN-γ capture assay and clonal analysis by limiting dilution

As we reported previously, we defined the relevant epitope and constructed HLA-B*35:01/NY-ESO-1 92–100 and 94–104 tetramers to quantify NY-ESO-1f-specific CD8+ T cells in PBMC [15]. Because the frequency of tetramer-positive cells ex vivo was below the limit of detection with the tetramer assay, CD8^+^ T cells were first stimulated in vitro with NY-ESO-1 peptide pools for 12 days. Under these conditions, tetramer-positive cells were detected in day 0 samples at frequencies of 0.1% of all CD8^+^ T cells for both the B*35:01/NY-ESO-1 92–100 and 94–104 tetramers (Table 3). They increased to 4.5% and 3.4%, respectively, at day 64 after vaccination. Although these values are somewhat lower than those obtained with the IFN-γ capture assay (Fig 1B), a similar pattern of dynamic changes of NY-ESO-1-specific CD8^+^ T cell frequency was seen in both assays. According to these results, we had previously concluded that NY-ESO-1f peptide vaccination successfully expanded antigen-reactive CD8^+^ T cells in patient TK-f01 and that these cells decreased again after repetitive vaccinations. In contrast, the present data on TCRB CDR3 sequences revealed that PBMCs contained low but readily detectable frequencies of NY-ESO1-specific CD8^+^ T cells before peptide vaccination, but that frequencies of these particular NY-ESO-1f-specific CD8^+^ T cells did not change on vaccination (Fig 4). Thus, the results of ex vivo TCRB CDR3 sequence analysis indicate that the frequency of NY-ESO-1-specific CD8^+^ T cells in the patient’s PBMC did not change during the course of vaccination (Fig 4 and Table 2). Our interpretation of these results is that the vaccine did not in fact increase the number of NY-ESO-1-specific CD8^+^ T cells, but imbued them with enhanced proliferation potential in the in vitro culture assays.

**Table 3:** The NY-ESO-1-specific CD8 T cell frequency (%) estimated by different assays.

				Day 0	Day 22	Day 43	Day 64	Day 106	Day 127	Day 148	Day 169	Day 211	Day 337
				Vac. 1	Vac. 2	Vac. 3	Vac. 4	Vac. 6	Vac. 7	Vac. 8	Vac. 9	Vac. 11	3 mo. Later
in vitro culture	IFN-γ capture assay *^1^			0.3	12.8	27.5	29.2	23.3	1	2.8	0.6	0.6	0.8
	Tetramer *^1^	NY-ESO-1 92–100	B*35:01	0.1			4.5		2.1	1.6	1.8	0.3	2.0
		NY-ESO-1 94–104	B*35:01	0.1			3.4		1.9	1.5	0.1	0.0	1.7
	Clonal analysis*^2^		B*52:01				0.0023						
	(Limiting Dilution)		B*35:01				0.0027						
ex vivo	TCR sequencing*^2^	6-8L TCRβ	B*52:01	0.7133	0.5291	0.6136	0.5079	0.7052	0.5572	0.5494			1.0550
		2F6 TCRβ	B*35:01	0.0000	0.0000	0.0000	0.0000	0.0007	0.0000	0.0000			0.0032

*^1^The numbers indicate the percentage of IFN-γ producing cells in cultured CD8^+^ T cells

*^2^The numbers indicate the frequency of clone in PBMCs (%).

We also estimated the NY-ESO-1f-reactive CD8^+^ T cell frequency after 12 day in vitro stimulation followed by limiting dilution (3 cells per well) (Table 3) [15]. By this method, the frequency of NY-ESO-1f peptide-reactive CD8^+^ T cells in PBMCs was estimated as 0.0027% (2.7×10^−5^) for B*35:01-restricted clones and 0.0023% (2.3×10^−5^) for B*52:01-restricted clones, assuming the doubling time of the cells to be 24 hr during the culture period. These results are quite similar to the TCRB CDR3 sequencing data in the case of the HLA-B*35:01-restricted CD8^+^ T cells. Thus, the frequency of B*35:01-restricted 2F6 sequences was 0.0007% (0.7×10^−5^) at day 106 and 0.0032% (3.2×10^−5^) at day 337 (Tables 2 and 3). However, the frequencies of HLA-B*52:01-restricted 6-8L TCRB sequences were 0.5291% to 1.0550% (0.5291×10^−2^ to 1.0550×10^−2^), much higher than those estimated by limiting dilution. Although the frequency of clone 2F6 sequences was low in PBMC, they might be efficiently expanded in vitro and detected by clonal analysis. In contrast, although clone 6-8L sequences were present at high frequencies in PBMC, they were perhaps less efficiently expanded by 12 day peptide stimulation and thus underestimated by this method. These results demonstrate the advantage of TCRB CDR3 sequence analysis that allows ex vivo direct detection and quantification of antigen-specific CD8^+^ T cells. In addition, anergic T cells that cannot produce IFN-γ in response to peptide stimulation and which are therefore undetectable by the IFN-γ capture assay, can be identified by TCRB sequencing.

### Limitations of TCR CDR3 sequencing using NGS in this study

A limitation of this strategy is that TCRB CDR3 sequences cannot provide any information regarding actual antigen specificity. Therefore, reference sequences for antigen-specific CD8 T cells are required for the analysis. In this study, we only sequenced two NY-ESO-1f-specific CD8^+^ T cell clones. We established several clones [15]; however, only limited numbers of clones could be maintained over long periods to be used for this study. IFN-γ-producing cells and tetramer-positive cells contain many different clones with similar specificities for NY-ESO-1f peptides, while we only analyzed HLA-B*35:01-restricted clone 2F6 and HLA-B*52:01-restricted clone 6-8L for TCR gene sequences. Therefore, many NY-ESO-1-specific CD8^+^ T cells may be missed, and the whole anti-NY-ESO-1 repertoire therefore underestimated. It would be desirable to use polyclonal NY-ESO-1f-reactive CD8^+^ T cells obtained by IFN-γ capture assay or tetramer staining instead of an assembly of clones for providing reference TCRB CDR3 sequences in future studies. Indeed, NGS can generate tremendous amounts of short sequence reads and profile TCR repertoires contained in the samples [7,23], that would enable us to catalog TCRB CDR3 sequences of antigen-specific T cells. In this way, heterogeneous DNA samples derived from polyclonal T cells could be analyzed by NGS without isolation of single DNA clones.

### Integrating NGS and other immune assays for immune monitoring

TCRB CDR3 sequences identified using NGS revealed that NY-ESO1-specific CD8^+^ T cells were present in the patient’s PBMCs before vaccination, but that peptide immunization induced no in vivo expansion of cells with these particular identified NY-ESO-1f-specific sequences (Fig 4). These T cells may have acquired enhanced proliferative potential after vaccination and more efficiently expanded in vitro when stimulated with antigenic peptide (Fig 1B). Such functional changes were only observed when monitoring by conventional immunological assays. These results are consistent with previous reports that melanoma patients who are being vaccinated may have already mounted a strong spontaneous T cell response against the types of antigens used in the vaccines. A spontaneous antitumor T cell response which has become ineffective may be reawakened by vaccination and can contribute to tumor rejection [24,25]. In addition, Valmori et al. reported that Melan-A peptide vaccination improved the functional avidity of Melan-A-specific T cells by the asynchronous expansion of several distinct T cell clones and selection of high-avidity T cells with increased tumor reactivity [26]. The advantages and disadvantages of currently available immunological assays for determining the frequencies of antigen-specific T cells are summarized in Table 4. NGS-based TCRB CDR3 identification integrated with other functional analyses should contribute to a better understanding of vaccine-induced immune responses and thus to the development of more effective cancer immunotherapies.

**Table 4:** Assays for evaluating antigen-specific T cell frequency.

Assay	Sensitivity	Advantages	Disadvantages
Tetramer MHC/peptide staining	10^−3^–10^−4^	- can evaluate epitope-specific T cells directly	- may not assess T cell function
			- has limited sensitivity
			- require in vitro re-stimulation
			- needs different kinds of tetramer for each HLA type or epitope peptide
Cytokine capture assay and CD107a assay	10^−3^–10^−4^	- is quantitative	- has limited sensitivity
		- can assess T cell function	- require in vitro re-stimulation
			- needs different kinds of antibody for each cytokine
ELISPOT	10^−4^–10^−5^	- is quantitative and sensitive	- has high background due to other cell activity
		- does not require in vitro re-stimulation	- requires reliable kits
		- can test a large number of samples simultaneously	- can evaluate limited kinds of cytokine
High-throughput sequencing (NGS)	10^−5^–10^−6^	- is quantitative and highly sensitive	- cannot evaluate antigen-reactive T cells directly
		- can assess TCR sequence comprehensively	- cannot assess T cell function

## Conclusions

TCRB CDR3 sequencing by NGS enables the identification of antigen-specific T cell clonotypes directly ex vivo and allows direct estimation of their frequencies. The ex vivo approach can avoid biases affected by cellular functions such as cell proliferation and cytokine production. Although peptide vaccination did not change the NY-ESO-1f-specific CD8^+^ T cell frequencies estimated by ex vivo TCRB CDR3 sequencing, the cells might gain enhanced capacity to proliferate in response to peptide stimulation in vitro, as demonstrated by IFN-γ capture assay and tetramer assay.

## Supporting Information

S1 Fig Reproducibility of TCRVB CDR3 analysis. To illustrate the reproducibility of the assay, we performed sequencing two separate PCR reactions performed using template from the same DNA extraction (Sample Day 337). Each point represents a single unique CDR3 sequence, plotted according to the relative frequency (%) in this log–log scatter plot. Clonotypes that were found in only one subset were assigned an arbitrary frequency value 0.0001 for graphical purposes. (Linear regression, R = 0.950, R^2^ = 0.902)
(PDF)

S1 Table TCRB CDR3 sequences in the PBMCs over the NY-ESO-1f peptide vaccine. CDR3 sequence >0.001% clones were listed.
(XLSX)
