# Supplementary material for: Detection and Tracking of NY-ESO-1-Specific CD8+ T Cells by High-Throughput T Cell Receptor β (TCRB) Gene Rearrangements Sequencing in a Peptide-Vaccinated Patient
Source: PLoS One. 2015 Aug 20;10(8):e0136086. doi: 10.1371/journal.pone.0136086 (PMC4546392; doi:10.1371/journal.pone.0136086)
Supplement: S1 Fig — To illustrate the reproducibility of the assay, we performed sequencing two separate PCR reactions performed using template from the same DNA extraction (Sample Day 337). Each point represents a single unique CDR3 sequence, plotted according to the relative frequency (%) in this log–log scatter plot. Clonotypes that were found in only one subset were assigned an arbitrary frequency value 0.0001 for graphical purposes. (Linear regression, R = 0.950, R2 = 0.902) (PDF) [file pone.0136086.s001.pdf]

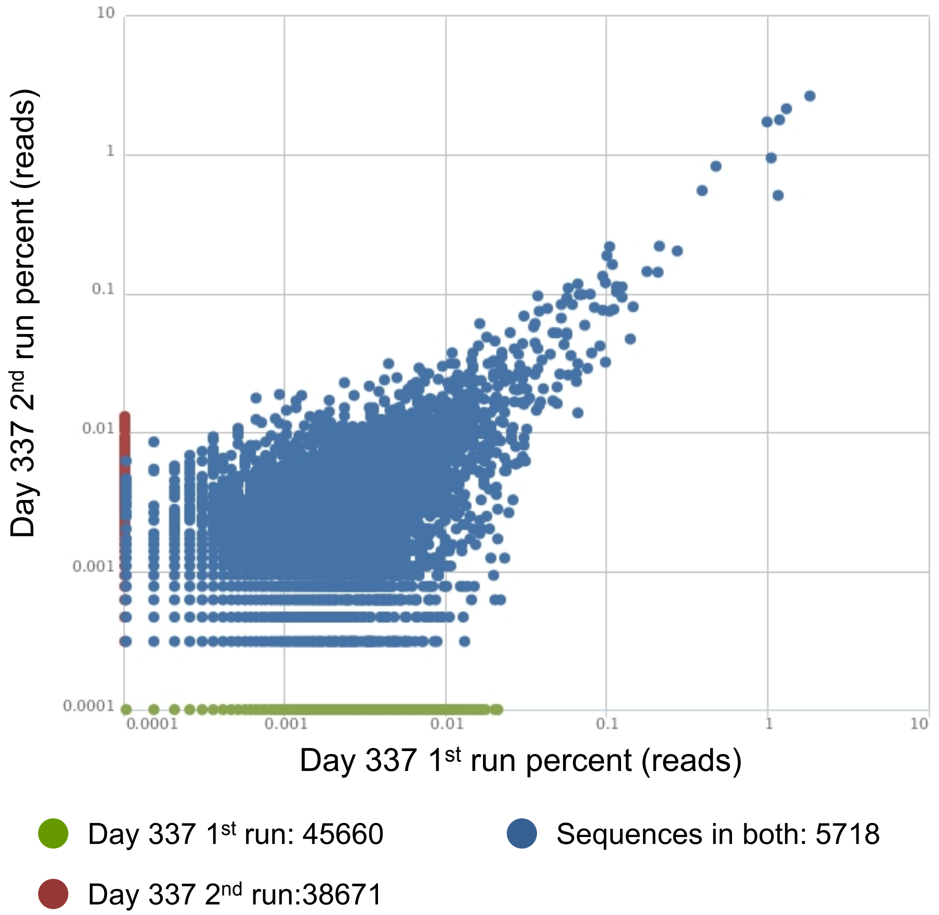

Supplementary Figure 1. Reproducibility of TCRVB CDR3 analysis. To illustrate the reproducibility of the assay, we performed sequencing two separate PCR reactions performed using template from the same DNA extraction (Sample Day 337). Each point represents a single unique CDR3 sequence, plotted according to the relative frequency (%) in this log–log scatter plot. Clonotypes that were found in only one subset were assigned an arbitrary frequency value 0.0001 for graphical purposes. (linear regression, R=0.950, R^2 = 0.902)
